# Trait impulsivity components correlate differently with proactive and reactive control

**DOI:** 10.1371/journal.pone.0176102

**Published:** 2017-04-19

**Authors:** Shihua Huang, Zude Zhu, Wei Zhang, Yu Chen, Shuangju Zhen

**Affiliations:** 1 School of Psychology, and Center for the Study of Applied Psychology, South China Normal University, Guangzhou, China; 2 School of Economic and Management, Guangzhou University of Chinese Medicine, Guangzhou, China; 3 School of Linguistics and Arts, and Collaborative Innovation Center for Language Competence, Jiangsu Normal University, Xuzhou, China; Centre de neuroscience cognitive, FRANCE

## Abstract

The relationship between impulsivity and cognitive control is still unknown. We hypothesized that trait impulsivity would differentially correlate with specific cognitive control processes. Trait impulsivity was measured by the Barratt Impulsiveness Scale, which assesses motor, attention, and non-planning impulsiveness components. Cognitive control was measured by a hybrid-designed Stroop task, which distinguishes proactive and reactive control. Thirty-three participants performed the Stroop task while they were scanned by functional magnetic resonance imaging. Proactive and reactive control involved increased activity in the fronto-parietal network, and brain activity was associated with impulsivity scores. Specifically, higher motor impulsiveness was associated with a larger proactive control effect in the inferior parietal lobule and a smaller reactive control effect in the right dorsolateral prefrontal cortex (DLPFC) and anterior cingulate contex. Higher attention impulsivity was associated with a smaller proactive control effect in the right DLPFC. Such a correlation pattern suggests that impulsivity trait components are attributable to different cognitive control subsystems.

## Introduction

Impulsivity is a component of personality concept that involves a tendency to display behavior characterized by little or no forethought, reflection, or consideration of the consequences. Therefore, difficulty in implementing cognitive control has been considered the key characteristic of people with high impulsivity [1]. Both substance and behavioral addictions, such as alcohol dependence [2] and gaming addiction [3], as well as schizophrenia [4], are characterized by reduced inhibitory control and increased self-reported impulsivity. However, the relationship between impulsivity and cognitive control is still largely unknown.

Previous studies have explored the relationship between impulsivity traits and cognitive control [5, 6]. One of the most widely used scales to measure impulsivity traits is the Barratt Impulsiveness Scale (BIS) [7]. The BIS includes three components: motor impulsiveness, attention impulsiveness, and non-planning impulsiveness. Some studies have reported correlations between the BIS total score and cognitive control measurements [8–10] while others have not [11, 12]. This discrepancy may have arisen due to the nature of the three individual impulsivity dimensions, which are modulatory and can correlate with each other. Indeed, differences have been found in studies testing correlations between cognitive control measures and individual dimensions of BIS [13].

For example, the correlation coefficient between motor impulsiveness and cognitive control has been found to be higher than that between the other two BIS dimensions and cognitive control, using cognitive control paradigms that included Go/No-Go, antisaccade, Stop Signal and Trail Making B [8, 14, 15]. In addition, Kam and colleagues [13] performed correlation analyses between BIS scores and event-related potentials (ERP) measured in the AX-CPT paradigm. Motor impulsiveness was linked with smaller P300 in general and larger N200 when conflict detection was involved. Non-planning impulsiveness was linked with smaller N200 when the inhibition of a primed response was required. Attention impulsivity was associated with inefficient conflict detection. These results thus suggest that the three BIS factors differentially correlate with cognitive control processes.

Just as impulsivity is comprised of different dimensions, cognitive control also includes a set of complex mental processes, and these subcognitive control systems may contribute to impulsivity traits differently. Braver and his colleagues [16] suggested a dual mechanism of cognitive control (DMC) framework, which postulates that cognitive control can be understood as operating via two primary modes: proactive and reactive. Proactive control provides relatively tonic maintenance of goal information [17–19], whereas reactive control acts as a flexible form of “late correction” in response to performance monitoring [16, 20]. Under the DMC framework, proactive and reactive control have been found to have a different neural basis, with sustained activation in the prefrontal cortex (PFC) contributing to proactive control, and the PFC, anterior cingulate cortex (ACC), posterior cortical, and medial temporal lobe regions contributing to reactive control. Such distinctions may map onto the dysfunction of a highly impulsive person. For example, previous studies suggested increased failure of proactive control and impaired reactive control in schizophrenia patients compared to healthy subjects [21, 22]. A systematic review of the literature suggests that patients with borderline personality disorder exhibit prefrontal dysfunctions across impulse components in orbitofrontal and dorsolateral PFC regions, whereas patients with attention-deficit/hyperactivity syndrome display disturbed activity mainly in the ventral lateral PFC and ACC [23].

Importantly, samples of people with schizophrenia and other mental disorders with or without a history of medication may produce results that are not representative of the general population. Thus, investigating the relationship between the subsystems of cognitive control and the impulsive trait in healthy adults will shed light on our understanding of impulsivity. However, few studies have examined trait impulsivity in relation to both reactive and proactive control.

The aim of the present study was to investigate the relationship between the impulsive trait and cognitive control, which were measured by the BIS and the Stroop task, respectively. The present study employed a blocked/event-related hybrid design of the Stroop task that has been successfully applied previously to disentangle proactive and reactive control processes. We predicted that BIS and cognitive control associations would be found in the fronto-parietal cognitive control network. Moreover, we hypothesized that this relationship might be varied across different dimensions of BIS and cognitive control subsystems.

## Materials and methods

### Participants

In total, 35 undergraduates participated in the study. However, two subjects were considered outliers on overall response time (above or below 2.5 standard deviations of the group mean). After eliminating these two participants, 33 undergraduates (15 females and 18 males; average age: 20 ± 1.2 years) yielded useable behavioral and imaging data. All of the participants were healthy, with no self-reported history of neurological or psychiatric disorders, and no use of psychiatric or cardiac medications. Participants had no sensorimotor deficits and had normal or corrected-to-normal visual acuity. Written informed consent was obtained from each subject and this study was approved by the Ethics Committee of Shenzhen People's Hospital, Shenzhen, China.

### Self-reporting questionnaires

We measured trait impulsivity with the Chinese version of the BIS [24]. By using exploratory and confirmatory factor analysis, previous studies have reported high reliability and validity of the BIS in college and community samples from the Chinese adult population [24–26]. This is a 30-item questionnaire that taps three subtraits: attention, motor, and non-planning impulsiveness. All items are answered to evaluate the occurrence of behavior using a five-point scale ranging from 1 (never) to 5 (always).

### Functional magnetic resonance imaging (fMRI) paradigm

A hybrid-design that incorporates block and trial events can be used to isolate proactive and reactive control [16]. Andrews-Hanna and colleagues [27] have successfully used a hybrid-designed Stroop task to investigate developmental trajectories of reactive and proactive control mechanisms across adolescence and early adulthood. Following the manipulation in Andrews-Hanna et al. [27], three types of trials (congruent, c; incongruent, i; neutral, n) were mixed in three types of blocks: Incongruent Blocks (I, including 50% incongruent trials and 50% neutral trials), Congruent Blocks (C, including 50% congruent trials and 50% neutral trials) and Neutral Blocks (N, 100% neutral trials). Participants were instructed to identify the font color of each word using one of four buttons on hand-held button boxes. On each trial, a color word (red, green, blue, or yellow) appeared for 1500 ms, followed by 500 ms of fixation between trials.

Half of the trials in each block consisted of stimuli that were specific to that block (i.e., i, c, n) and the remaining half of the trials consisted of neutral stimuli that appeared across all blocks. The trial types within blocks were pseudo-randomly ordered such that no more than two trials of the same type could appear in a row. Hence, within the congruent blocks, six congruent trials (Cc) were mixed with six block-general neutral trials (Cn) to allow for comparisons between trial types within blocks. Similarly, within incongruent blocks, six incongruent trials (Ii) were mixed with six block-general neutral trials (In). Neutral blocks consisted of six neutral trials that were specific to the neutral block and six block-general neutral trials (Nn). In total, participants completed 324 task trials, with 54 trials corresponding to each trial type. The experiment was divided into three runs. Each run comprised four, 30 s fixation (F) blocks interleaved with nine, 45 s task blocks. The order of the three runs was: F-C-I-N-F-I-N-C-F-N-C-I-F, F-I-N-C-F-N-C-I-F-C-I-N-F, and F-N-C-I-F-C-I-N-F-I-N-C-F.

### MRI data acquisition

MRI data were acquired using a Siemens Trio 3T MRI system with an eight-channel head coil at the Shenzhen Institutes of Advanced Technology, Chinese Academy of Science. Functional MRI data were collected with EPI sequence, slice number = 33, matrix size = 64*64, FOV = 220*220mm, TR/TE = 2000/30ms, FA = 90 º, slice thickness = 3.5 mm, and gap = 0 mm. T1-weighted high-resolution structural images were acquired using a magnetization-prepared rapid acquisition gradient echo sequence (176 slices, TR = 1900 ms, TE = 2.53 ms, FA = 9 º, voxel size = 1 × 1 × 1 mm^3^).

### Data analysis

#### Behavioral data analyses

Group comparisons were conducted to reveal the performance difference of the Stroop task between groups. Binary and partial correlations were conducted to test the relationship between BIS and the performance in the Stroop task. Unless otherwise noted, the significance of statistical tests (e.g., paired t-tests, independent samples t-tests, correlation analyses) was set using a two-tail comparison.

#### fMRI analyses at the subject level

Statistical Parametric Mapping (SPM 8; Welcome Department of Cognitive Neurology, UCL, London, UK) was used in the preprocessing and statistical analyses of the fMRI data. Differences in timing between slices of the raw data were adjusted using sync interpolation. The timing-corrected images were then realigned to the first volume in order to correct for head motion. The functional images were then co-registered to native high-resolution T1-weighted images and transformed into Montreal Neurological Institute 2 × 2 × 2 mm standard space with an 8 mm full-width at half-maximum Gaussian kernel. Finally, high-pass filtering (with a 128 s cutoff) was applied to the images to remove low-frequency drifts.

As described previously, the hybrid block/event-related task paradigm was designed such that block effects and event-related effects would be modeled within separate general linear models (GLMs). Similar to previous study [27], one GLM was used to define block effects. The GLM included a regressor for each block type: congruent (C), incongruent (I), neutral (N) and fixation (F) blocks. For each regressor, a double-gamma response function was convolved with the onset of each trial. The proactive control was defined as I-N (i.e. the Stroop interference effect).

For reactive control, we improved the methods used in Andrews-Hanna et al’s study [27]. Specifically, the trial effect was nested in the block effect; we thus added block regressors in order to isolate the trial type effect. That is, in addition to the four trial type regressors, a block regressor that combined four block types was also included in the GLM. For each regressor, a double-gamma response function was convolved with the onset of each trial. The reactive control was defined as Ii—In (i.e., the trial-related Stroop interference effect).

#### fMRI analyses at group level

For the proactive control effect, group analyses compared activation differences in I and N blocks. For the reactive control effect, group analyses compared activation differences in Ii and In conditions.

Monte Carlo simulations using the revised AlphaSim program were used to determine the appropriate combination of the significance level and cluster threshold required to reach a corrected significance level of *p* < 0.05, taking into account both native space voxel dimensions and the effective smoothness estimated directly from our preprocessed data (https://afni.nimh.nih.gov/pub/dist/doc/program_help/3dClustSim.html). The Monte Carlo simulations used 1,000 iterations and indicated a significance level of *p* < 0.005 and cluster threshold of 80 voxels in order to reach a corrected significance level of *p* < 0.05. This threshold was applied to each of the contrasts described in the study.

#### Region of interest (ROI) analyses and correlation analyses

To test the relationship between impulsivity and cognitive control, ROI analyses were conducted. Separate spherical ROIs with an 8 mm radius were created based on the peak coordinates of the brain regions revealed by the above mentioned proactive and reactive control contrast. Such an ROI construction approach is widely used [28] to ensure each ROI has the same number of voxels. The beta estimates of all the conditions involved in the proactive and reactive control contrast were then extracted for each participant within each ROI using the MarsBar toolbox.

To examine whether the subsystems of cognitive control explained impulsivity, partial correlation analyses between the activation magnitude of proactive and reactive control and the score of each impulsivity dimension were conducted with and without controlling for the other impulsivity dimensions, age and sex. The residuals of variables in the partial correlation were used in the scatter plots. A boxplot was used to detect outliers before correlation analyses.

## Results

### Behavioral data

The mean BIS composite score was 66.31 ± 9.26 (mean ± SD). The mean subscale scores were 23.39 ± 4.24 for attention impulsiveness, 21.24 ± 4.0 for motor impulsiveness, and 21.68 ± 4.46 for non-planning impulsiveness.

Consistent with prior studies, response time and accuracy in the Stroop color-word task significantly differed across conditions, when examined in both a blocked and in a trial-by-trial fashion (Table 1). As would be expected, there were robust Stroop interference effects. Participants were significantly slower and less accurate when responding to incongruent blocks (I) than to neutral blocks (N) (Reaction Time [RT] Effects, *t* (32) = 5.57, *p* < 0.001; Accuracy Effects, *t* (32) = -2.47, *p* < 0.05). Additionally, participants were significantly slower and less accurate when responding to incongruent trials (Ii) than to neutral trials within incongruent blocks (In) (RT Effects, *t* (32) = 8.97, *p* < 0.001; Accuracy Effects, *t* (32) = -4.19, *p* < 0.001).

**Table 1:** Mean (standard deviation) of response time and accuracy for the proactive and reactive control related conditions.

	Response Time (ms)	Accuracy (%)
Incongruent Blocks (I)	754 (118)	88 (9)
Neutral Blocks (N)	712 (108)	90 (10)
Incongruent trials in I blocks (Ii)	793 (135)	86 (10)
Neutral trials in I blocks (In)	714 (107)	90 (9)

Bivariate correlations between behavioral performances and BIS scores were calculated. For proactive control, the motor score and attention score were not significantly correlated with the RT effect or accuracy effect (*ps* > 0.15). However, the larger RT interference effect was associated with a higher non-planning score (*r* = 0.36, *p* < 0.05) and remained significant when the other BIS subscale scores, age and sex were controlled (*r* = 0.57, *p* < 0.01). For reactive control, the motor score and attention score also did not significantly correlate with the RT effect or accuracy effect (*ps* > 0.15). However, the larger RT interference effect was associated with a higher non-planning score (*r* = 0.35, *p* < 0.05) and remained significant when the other BIS subscale scores, age and sex were controlled (*r* = 0.57, *p* < 0.01).

### Imaging results

#### Proactive control effect

**Fig 1:**
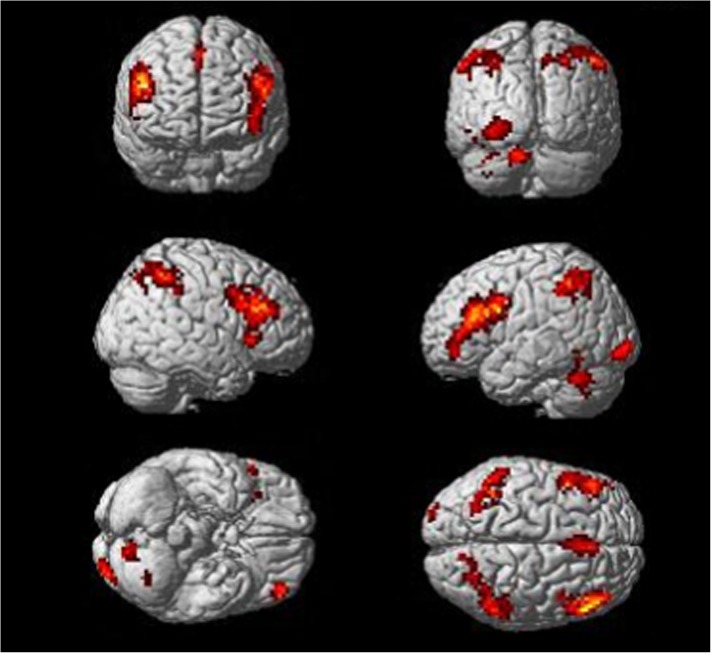
Significant brain regions for proactive control (Incongruent-Neutral blocks) in all participants.

Higher brain signal in the I blocks than the N blocks was found in the dorsolateral prefrontal cortex (DLPFC), ACC, inferior parietal lobule (IPL), and inferior occipital gyrus on both sides of the brain (Table 2 and Fig 1).

**Table 2:** Significant brain regions for proactive control (Incongruent–Neutral blocks).

	Voxels	x	y	z	Peak T
DLPFC (L)	502	-42	8	28	7.1
DLPFC (R)	467	48	35	28	7.0
IPL (L)	198	-42	-49	46	6.6
IPL (R)	195	51	-49	49	6.3
ACC (B)	120	6	32	43	4.8

Note: R, right; L, left; B, bilateral; DLPFC = Dorsolateral prefrontal cortex; IPL = Inferior Parietal Lobule;ACC = Anterior Cingulate Cortex.

**Fig 2:**
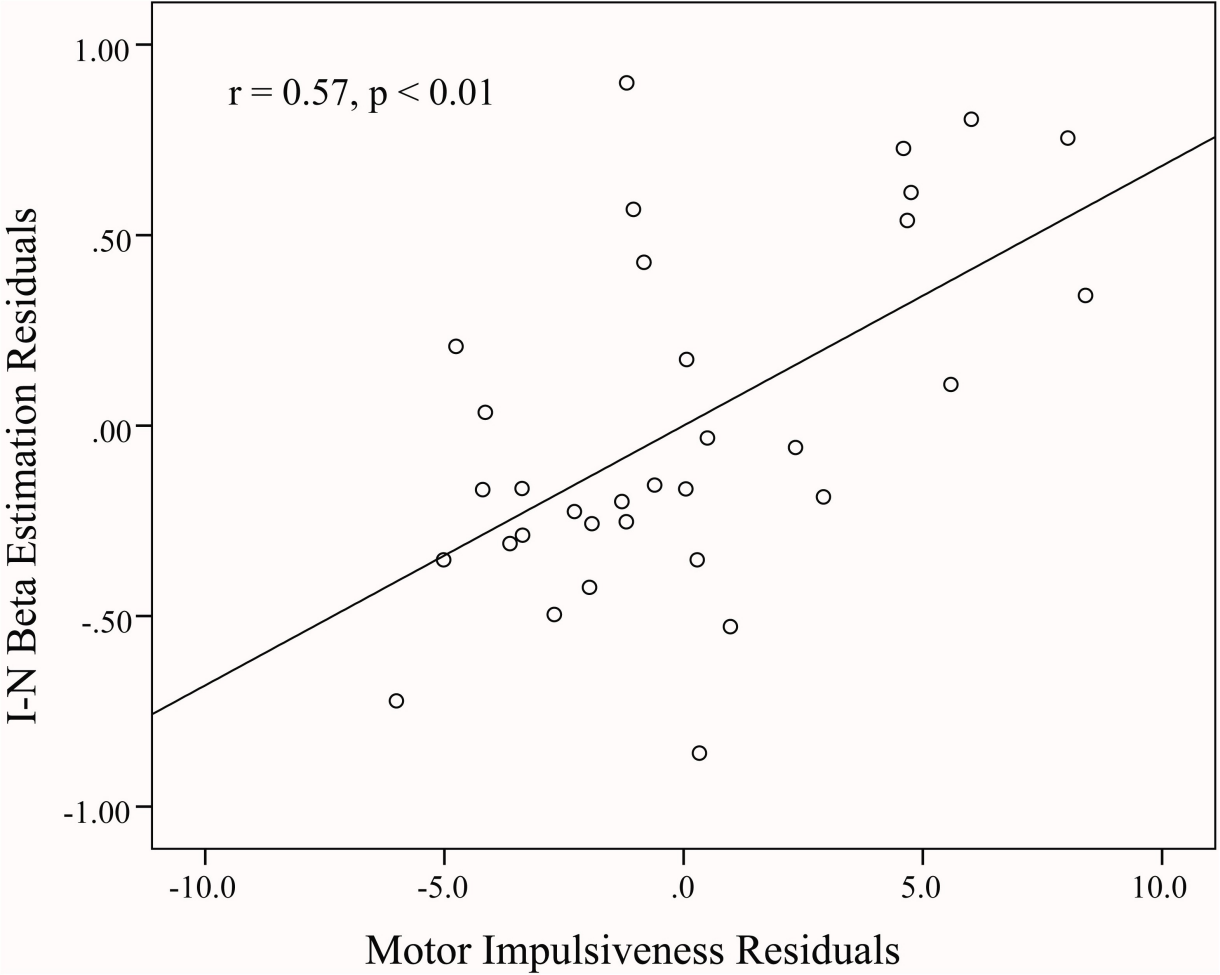
Partial correlation between proactive control related activity and self-report measure of motor impulsiveness, after controlling for age, sex, attentional and non-planning scores. Residuals were used in the scatter plot. Beta estimation (percent signal change) (Incongruent—Neutral blocks) was extracted from the left IPL.

**Fig 3:**
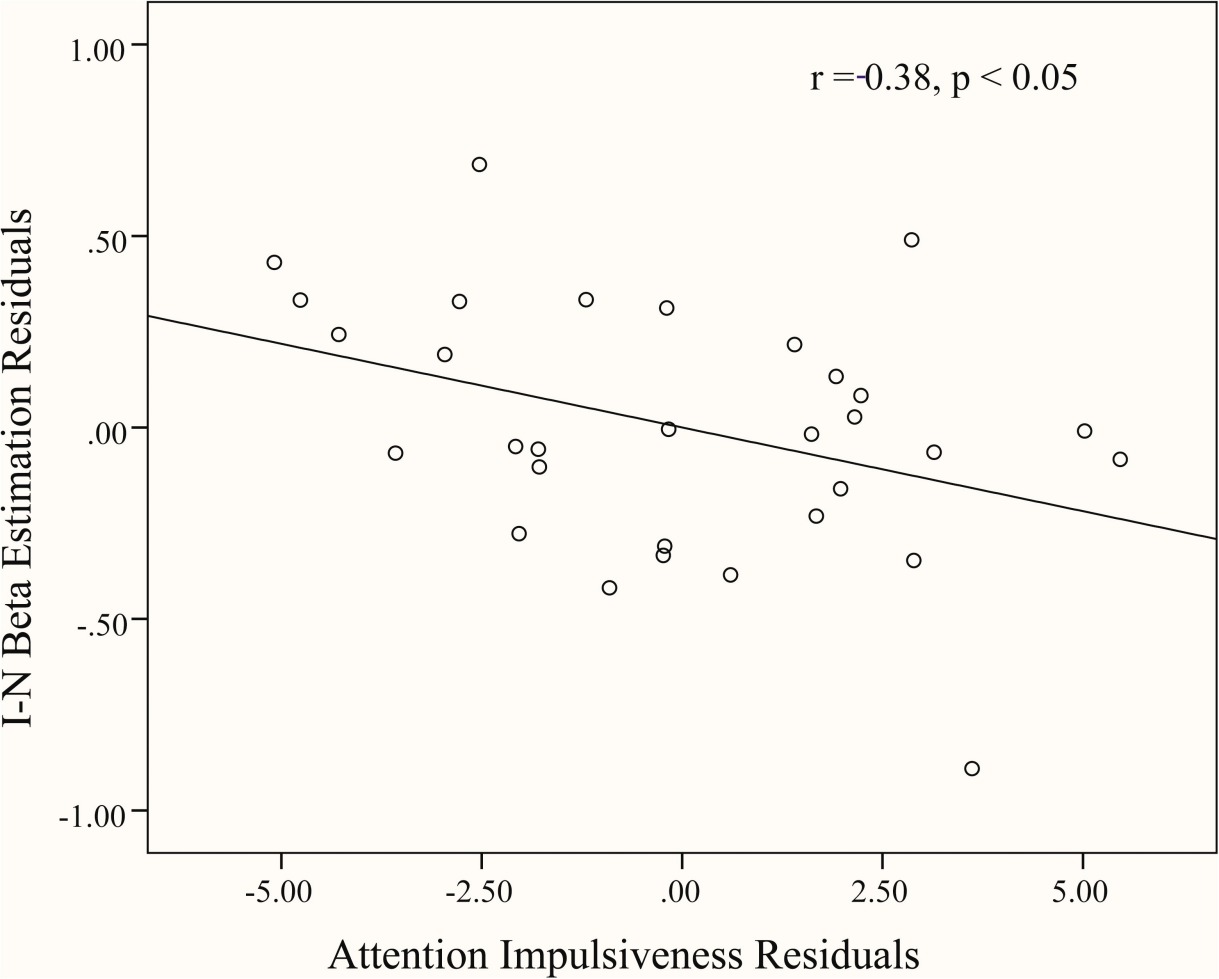
Partial correlation between proactive control related activity and self-report measure of attention impulsiveness, after controlling for age, sex, non-planning and motor scores. Residuals were used in the scatter plot. Beta estimation (percent signal change) (Incongruent—Neutral blocks) was extracted from the right DLPFC.

To uncover the brain-neuropsychological relationship, brain signals (I block–N block) in the DLPFC, IPL, and ACC were entered in correlation analyses. The results of correlation revealed that, the BIS and non-planning scores were not significantly correlated with brain signals in any region. However, motor impulsiveness was positively correlated with brain signal in left IPL (*r* = 0.47, *p* < 0.01), and the significance remained after the other two BIS subscale scores, age and sex were controlled (*r* = 0.57, *p* < 0.01) (Fig 2). Attention impulsiveness also was significantly correlated with brain signals in right DLPFC with (*r* = -0.38, *p* < 0.05) and without the two other BIS subscale scores, age and sex being controlled for (*r* = -0.33, *p* = 0.07) (Fig 3).

#### Reactive control effect

**Fig 4:**
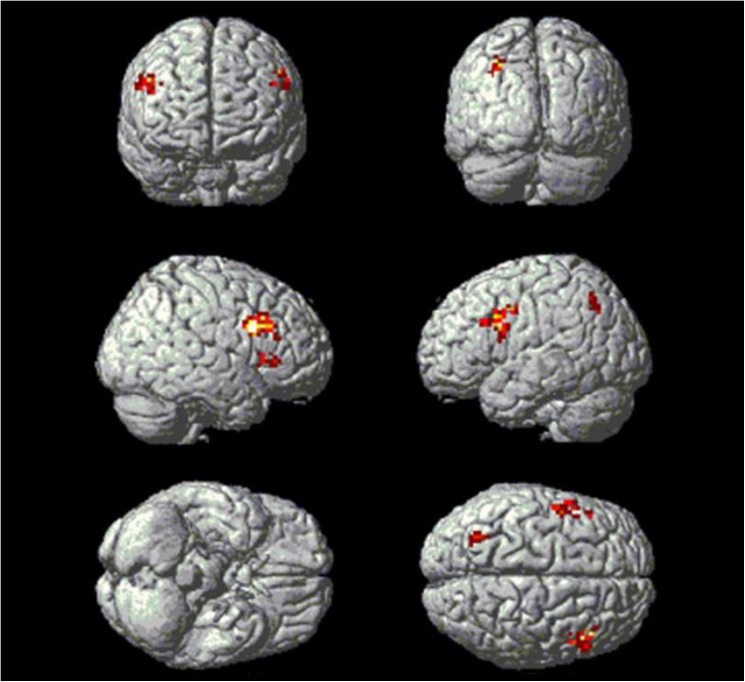
Significant brain regions for trial-related fMRI Stroop activity (Ii-In) in all participants. Note: *Ii*, *incongruent trials in Incongruent blocks; In*, *neutral trials in Incongruent blocks*.

The contrast between incongruent trials (Ii) and neutral trials (In) within the incongruent blocks revealed significant activation in bilateral DLPFC (Table 3 and Fig 4).

**Table 3:** Significant brain regions for reactive control (Ii-In).

	Voxels	x	y	z	Peak T
DLPFC (R)	105	45	14	28	5.09
DLPFC (L)	76	-54	-4	43	4.99

Note: L, left; R, right; DLPFC = Dorsolateral prefrontal cortex. Ii, incongruent trials in Incongruent blocks; In, neutral trials in Incongruent blocks.

**Fig 5:**
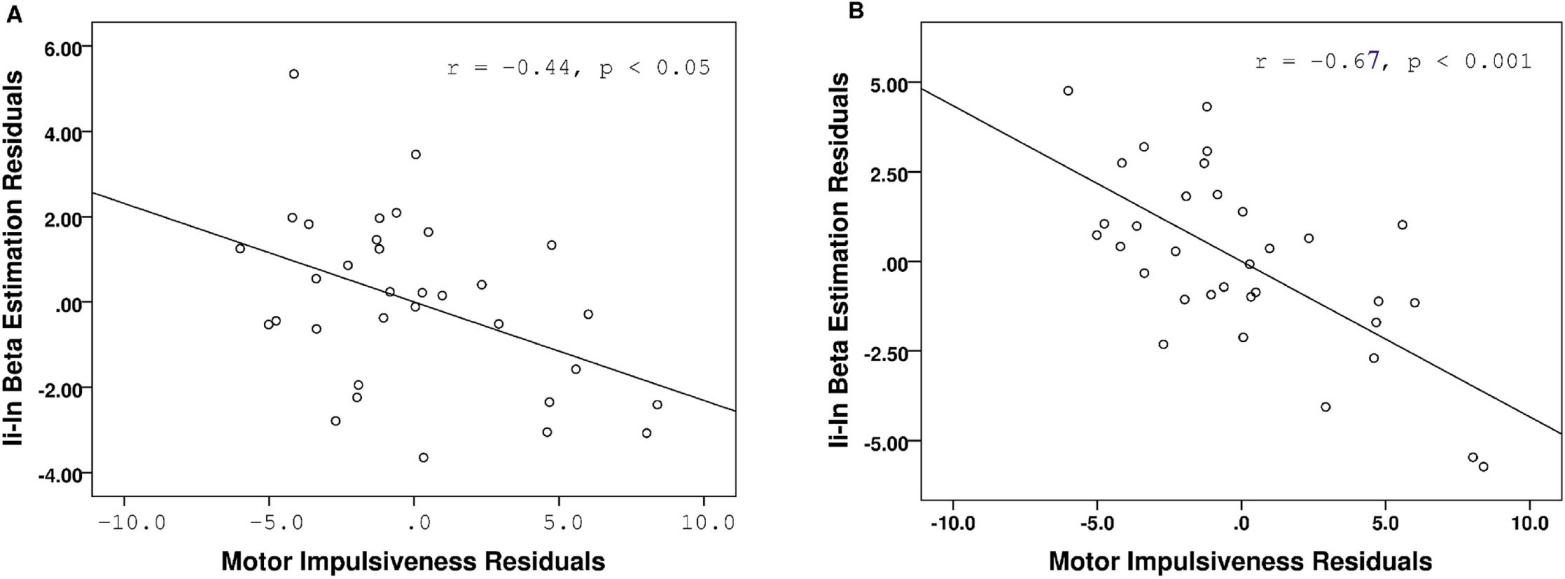
Partial correlation between reactive control activity and self-report measure of motor impulsiveness, after controlling for age, sex, attentional and non-planning scores. Residuals were used in the scatter plot. Beta estimation (percent signal change) in the contrast of Ii vs. In were extracted from the right DLPFC (A) and ACC (B).

In the brain-neuropsychological correlation analysis, brain signals in the DLPFC and ACC were extracted. The ACC was included as an ROI because the region has been shown to play an important role in reactive control although no significant activation was found in the present contrast. Correlations revealed that motor impulsiveness was negatively correlated with brain signal in the right DLPFC (*r* = -0.42, *p* < 0.05), and the significance remained when the other two BIS subscale scores, age and sex were controlled (*r* = -0.44, *p* < 0.05) (Fig 5A). Interestingly, the brain signal in the ACC was also significantly correlated with motor impulsiveness (*r* = -0.66, *p* < 0.001), and the significance remained when the other two BIS subscale scores, age and sex were controlled (*r* = -0.67, *p* < 0.001) (Fig 5B).

## Discussion

To investigate the correlation between cognitive control and impulsivity, the present study adopted a mixed design in the Stroop paradigm to isolate proactive and reactive control and examined their correlations with BIS scores. Although not correlated with composite BIS scores, the proactive and reactive control related interference effects significantly correlated with BIS subscale scores. Specifically, during proactive control, attention impulsiveness was correlated with brain activity in the right DLPFC, and motor impulsiveness was correlated with brain activity in the left IPL. During reactive control, motor impulsiveness was correlated with brain activity in both the right DLPFC and the ACC. The results provide insights for understanding the relationships between cognitive control and impulsivity traits.

Inconsistent results were found when investigating the relationship between cognitive control and BIS in previous studies [8, 9, 11, 12]. While some studies found correlations between cognitive control and BIS scores, other studies found no such correlation. One reason for this discrepancy may be the heterogeneity of the BIS subscales and the complexity of the cognitive control processes. Indeed, when the subscale scores were used in our analyses, the results revealed significant correlations between impulsivity and cognitive control, in line with results in a recent ERP study [13]. Importantly, the present study extended previous results in two ways. First, we investigated not only the subscales of the BIS but also subcognitive control processes (i.e., proactive and reactive control). Secondly, the present study investigated the correlations between BIS subscale scores and brain activation during proactive and reactive control.

Significant brain activation in the fronto-parietal network, including regions of DLPFC, IPL, and ACC on both sides of the brain, was found during proactive control. These results were in line with previously published findings on the brain regions engaged in proactive control [16, 20, 29]. Moreover, a significant negative correlation between activity in the right DLPFC and attention impulsiveness was found. The DLPFC is thought to proactively bias attention towards task-relevant goals and representations [30]. Previous studies found that highly impulsive individuals might manifest a decreased sustained brain activity in lateral prefrontal regions including the DLPFC [27, 31–33], results that are consisted with supported the current negative correlation between DLPFC activation and attention impulsiveness found in the current study.

The results revealed a significant proactive control effect in the IPL on both sides of the brain, with increased brain activity associated and higher motor impulsiveness. This result is in agreement with previous findings showing that gray matter volume in the IPL is negatively correlated with extraversion [34]. Activation in the IPL was also found in previous Stroop studies [4, 35] and was associated with working memory, which is important for proactive control [36–38]. In the present study, people with higher motor impulsiveness may have had difficulty in completing proactive control processes, which enhanced activation in the IPL. The left IPL may be involved in representing and comparing conflict information to serve the proactive goal. Attention impulsiveness was associated with the right DLPFC, a region that has been previously found to be activated during the Stroop task, the stop-signal task, and the Go/NoGo task [39]. Activation of the right DLPFC plays important roles in sustained attention [40], especially attention selection in an inteference information context [41] and during response inhibition [42].

For reactive control, the results revealed significant brain activation in the DLPFC on both sides of the brain, in line with previous results [16, 20, 29]. Reactive control, which reflects flexibility in transient event change or monitoring, is associated with brain activity in both the DLPFC and ACC [16, 43]. Interestingly, the present study revealed significant reactive control effects in the DLPFC but not the ACC. However, as ACC activation was found during proactive control, the lack of activation in the ACC during reactive control does not appear to be due to a lack of statistical power or inappropriate task design. Moreover, a significant correlation between activation in the ACC during reactive control and BIS score was found. Specifically, motor impulsiveness was significantly correlated with the magnitude of brain activity in the ACC (*r* = -0.44), which is similar to the correlation between motor impulsiveness and brain activity observed in the DLPFC (*r* = -0.67). These results suggest that higher motor impulsiveness was associated with a smaller change in brain activity during reactive control.

By incorporating proactive and reactive control, the present study extends previous results by showing unbalanced correlations between impulsivity components and cognitive control subsystems. Motor and attention impulsiveness were correlated with both proactive and reactive control, while non-planning impulsiveness was only correlated with behavioral measurements. Unbalanced correlations between cognitive control and BIS components [44, 13] or different impulsivity scales [11, 45] were reported previously. Attention and motor impulsiveness showed consistent correlations with cognitive control subsystems.

Attention and motor impulsiveness were highly correlated with cognitive control. These results are consistent with previous studies that have suggested that brain regions involved in cognitive control include the DLPFC, especially the right DLPFC [46, 47]. For example, response inhibition, a key component of cognitive control, has been associated with significant activation of the right DLPFC in healthy volunteers [42], whereas dysfunctional activity in the right DLPFC during response inhibition has repeatedly been reported as a prominent hallmark in drug addiction [48]. More recently, hypo-activation in the right DLPFC in adult attention-deficit/hyperactivity syndrome was specifically related to impaired response inhibition [49]. Hence, although the present results cannot be generalized to any clinical population directly, they suggest the potential utility of such paradigms (i.e., assessing multiple cognitive control measurements relative to individual impulsivity traits) in impulsivity disorders or other psychiatric disorders with increased trait impulsivity (e.g., substance abuse disorders and eating disorders).

The unbalanced correlation pattern might also explain the correlation between non-planning impulsiveness and behavioral indices but not brain activity during cognitive control. Previous studies [45] have found that the non-planning score was associated with brain activity in the striatal region, which is not a typical cognitive control region in the Stroop task. The non-planning score is considered an index of temporal impulsivity and may thus differ from attention and motor impulsiveness.

There are limitations in the present study. First, although the activation found in DLPFC for reactive control suggest the design has enough power to detect reactive control related brain activity, we did not found significant activation in the ACC. Given the ACC may initially monitor conflict during cognitive control [50], it might need enough of an inter-trial interval to isolated the monitoring and later-on processes. Future studies are required to test this possibility. Second, only a Stroop task was used to measure cognitive control; future studies may use multiple cognitive control paradigms to better target the complex relationship between cognitive control subsystems and BIS components. Third, a recent study [51] has suggested two dimension structure for BIS (cognitive impulsivity, behavioral impulsivity) based on the western cultural context. It would be interesting to test whether the two dimensions are correlated with cognitive control subsystems differently and if the two dimensions structure would be validated in Chinese sample.

In conclusion, by employing the DMC framework to target proactive control and reactive control on the one hand, and measuring impulsivity using the subscales of the BIS on the other hand, the present study revealed that motor impulsiveness was correlated with both proactive and reactive control systems. The results suggest that specific impulsivity components have different associations with cognitive control subsystems.

## Conclusions

1. Proactive and reactive control involved increased activity in the fronto-parietal network, and brain activity was associated with impulsivity scores.
2. Higher motor impulsiveness was associated with a larger proactive control effect in the left inferior parietal lobule and a smaller reactive control effect in the right dorsolateral prefrontal cortex (DLPFC) and anterior cingulate cortex.
3. Higher attention impulsivity was associated with a smaller proactive control effect in the right DLPFC.

## Supporting information

S1 Table Relevant behavioral and fMRI data underlying the findings described in manuscript. Cc: congruent trials; Cn: neutral trials within congruent blocks; Ii: incongruent trials; In: neutral trials within incongruent blocks; in_trail: Trial Interference = Ii-In.
cn_trail: Trial Facilitation = Cc-Cn; IN_block: Blocked Interference = Incongruent Blocks—Neutral blocks; CN_blcok: Blocked Facilitation = Congruent Blocks—Neutral blocks.
(XLSX)

## Abbreviations

ACC: anterior cingulate cortex
BIS: Barratt Impulsiveness Scale
DLPFC: dorsolateral prefrontal cortex
DMC: dual mechanism of cognitive control
fMRI: functional magnetic resonance imaging
IPL: inferior parietal lobule
PFC: prefrontal cortex
ROI: region of interest
RT: reaction time
SD: standard deviation
